# Real-Time MRI Guidance for Reproducible Hyperosmolar Opening of the Blood-Brain Barrier in Mice

**DOI:** 10.3389/fneur.2018.00921

**Published:** 2018-10-26

**Authors:** Chengyan Chu, Guanshu Liu, Miroslaw Janowski, Jeff W. M. Bulte, Shen Li, Monica Pearl, Piotr Walczak

**Affiliations:** ^1^Division of MR Research, Russell H. Morgan Department of Radiology and Radiological Science, The Johns Hopkins University School of Medicine, Baltimore, MD, United States; ^2^Cellular Imaging Section and Vascular Biology Program, Institute for Cell Engineering, The Johns Hopkins University School of Medicine, Baltimore, MD, United States; ^3^Neurology Department, Dalian Municipal Central Hospital Affiliated with Dalian Medical University, Dalian, China; ^4^F. M. Kirby Research Center, Kennedy Krieger Research Institute, Baltimore, MD, , United States; ^5^NeuroRepair Department, Mossakowski Medical Research Centre, Polish Academy of Sciences, Warsaw, Poland; ^6^Department of Neurosurgery, Mossakowski Medical Research Centre, Polish Academy of Sciences, Warsaw, Poland; ^7^Division of Interventional Neuroradiology, Russell H. Morgan Department of Radiology and Radiological Science, The Johns Hopkins University School of Medicine, Baltimore, MD, United States; ^8^Department of Neurology and Neurosurgery, Faculty of Medical Sciences, University of Warmia and Mazury, Olsztyn, Poland

**Keywords:** blood brain barrier, intra-arterial, mannitol, MRI, mouse model

## Abstract

The blood-brain barrier (BBB) prevents effective delivery of most therapeutic agents to the brain. Intra-arterial (IA) infusion of hyperosmotic mannitol has been widely used to open the BBB and improve parenchymal targeting, but the extent of BBB disruption has varied widely with therapeutic outcomes often being unpredictable. In this work, we show that real-time MRI can enable fine-tuning of the infusion rate to adjust and predict effective and local brain perfusion in mice, and thereby can be allowed for achieving the targeted and localized BBB opening (BBBO). Both the reproducibility and safety are validated by MRI and histology. The reliable and reproducible BBBO we developed in mice will allow cost-effective studies on the biology of the BBB and drug delivery to the brain. In addition, the IA route for BBBO also permits subsequent IA delivery of a specific drug during the same procedure and obtains high targeting efficiency of the therapeutic agent in the targeted tissue, which has great potential for future clinical translation in neuro-oncology, regenerative medicine and other neurological applications.

## Introduction

The treatment efficacy for many central nervous system (CNS) diseases is hindered by limited access to therapeutic agents. The poor drug penetration is mainly caused by the blood-brain barrier (BBB), which sequesters the CNS from the systemic circulation. As a consequence, more than 98% of the pharmaceutical agents do not enter the brain after intravascular delivery (1–3). Thus, strategies are needed that will safely and efficiently disrupt the BBB. Intra-arterial (IA) mannitol followed by the infusion of therapeutic agents, including chemotherapeutics, gene vectors, and stem cells, has been the primary method by which therapeutics have been delivered across a disrupted BBB for several decades, both in preclinical models and in clinical studies (4–8). However, osmotic BBB opening (BBBO) proved highly variable and inconsistent (9), as it is affected by multiple factors, including the mannitol dose, injection speed, vascular anatomy, and cerebral hemodynamics (10). This complexity and the inconsistent outcomes have resulted in highly variable published protocols (11–14).

The vast majority of previous preclinical reports on BBBO used rats or larger animals, because of the technical challenges that are encountered in mice. Our motivation to establish a reliable and safe protocol for BBBO in mice was that mice are low cost and are used for the majority of disease modeling studies. There is also an abundance of transgenic mice, indispensable for gaining insights into a variety of disorders (15, 16). Furthermore, our previous IA injection experiments in several species (10, 17–19) have shown the value and importance of monitoring local trans-catheter perfusion with MRI, allowing real-time adjustment of infusion parameters for precise and predictable BBBO and/or delivery of therapeutic agents at physiologically relevant, non-damaging infusion rates. We report here on a safe and reproducible technique to disrupt the local BBB in mice under the guidance of interventional MRI. We believe that, with our approach, the method to achieve BBBO can be reconsidered and can be re-established as a precise and effective technique that can facilitate drug delivery to the brain.

## Materials and methods

### Anesthesia and carotid artery catheterization

All procedures were approved by The Johns Hopkins Animal Care and Use Committee. Male SCID mice (6–8 weeks old, 20–25 g, Jackson Laboratory) were anesthetized with 2% isoflurane. The common carotid artery (CCA) bifurcation was exposed and the occipital artery branching off from the external carotid artery (ECA) was coagulated. The ECA and the pterygopalatine artery (PPA) were temporarily ligated with 4–0 silk sutures. A temporary ligature using a 4–0 suture was placed on the carotid bifurcation and the proximal CCA was permanently ligated. A microcatheter (PE-8-100, SAI Infusion Technologies) was flushed with 2% heparin (1,000 units per ml, heparin sodium, Upjohn), inserted into the CCA via a small arteriotomy and advanced into the internal carotid artery. Before cannulation, a droplet of glue was added to the outer surface of the catheter to tightly ligate the catheter to the vessel.

### Interventional MRI

The mice with a secured intra-arterial catheter were positioned in a Bruker 11.7T MRI scanner. Baseline T2 (TR/TE = 2,500/30 ms) and T1 (TR/TE 350/6.7 ms)-weighted and dynamic Gradient echo-echo planar imaging (GE-EPI, TR/TE 1250/9.7 ms, field of view (FOV) = 14, matrix = 128, acquisition time = 60 s and 24 repetitions) images of the brain were acquired. A superparamagnetic iron oxide (SPIO) nanoparticle formulation (Feridex®, dissolved in saline at 1:30; 0.3 mg Fe/ml) was infused intra-arterially at rates between 0.05 and 0.20 ml/min under dynamic GE-EPI MRI to predict perfusion territory and optimize that territory to the desired size and location. An infusion pump (PHD 2000, Harvard Apparatus Inc.) was used to control SPIO administration. The routine was to start injections from the lowest speed, and then increase it at increments of 0.05 ml/min until an effective perfusion area was achieved. Then, 25% mannitol was delivered via an IA route over 1 min at a speed determined by previous contrast agent injection. Five minutes after mannitol injection, mice received 0.07 ml of gadolinium (Gadoteridol, 279.3 mg/mL) intraperitoneally. T1-weighted images were obtained post-gadolinium to visualize BBB integrity (20).

### Histological validation of BBBO

For histological evaluation of BBBO, 0.1 ml of IA 2% w/v Evans blue (EB) or rhodamine red (1 mmol/l) were subsequently administrated intra-arterially at the same rate as mannitol. The brains were harvested right after injection and without perfusion to avoid the clearance of the both imaging agents. For EB verification, frozen coronal brain slabs (1-mm) were sliced on a cryostat. For the detection of rhodamine, the brain was cryosectioned at 30 μm and fluorescent images of rhodamine biodistribution were acquired.

### Immunohistochemistry to evaluate the safety of BBBO

Seven days after BBBO, animals were transcardially perfused with Five percent sucrose and then with Four percent paraformaldehyde. The brains were cryopreserved in 30% sucrose and cryosectioned at 30-μm. Primary antibodies and dilutions were used as follows: anti-GFAP (1:250, Dako); anti-Iba1 (1:250, Wako); and anti-NeuN (1:100, Cell Signaling Technology). The secondary antibody was goat anti-rabbit (Alexa Fluor-488, 1:200, Molecular Probes).

### Image processing and statistical analysis

Quantitation of immunohistochemistry results was based on relative fluorescence using Image J and analyzed with Mann-Whitney *U*-test. While stereology is more accurate and appropriate when absolute cell numbers are essential, in case of microglial or astrocytic activation both cell number and signal intensity are relevant, thus we measured relative fluorescence. The MRI analysis of the change in area of the SPIO perfusion territory and Gd-enhancement for each mouse was calculated by a custom-written script in MATLAB and also analyzed with Mann-Whitney *U*-test. A Pearson correlation coefficient comparing the above-mentioned areas was calculated using GraphPad software.

## Results

### Real-time MRI for prediction of trans-catheter perfusion territory using SPIO

**Figure 1:**
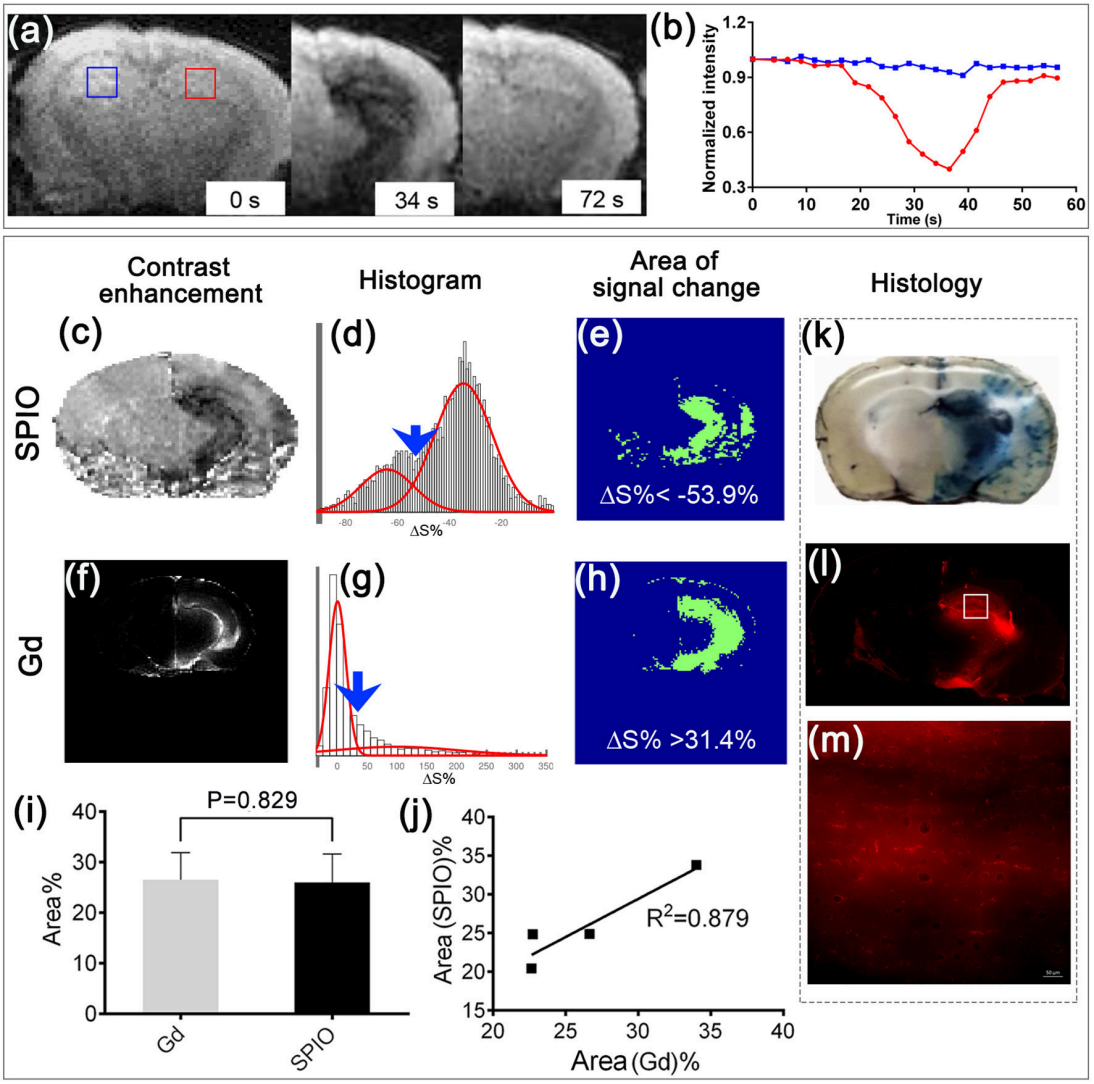
Real-time MRI to predict BBBO territory and histological validation. **(a)** Representative T2^*^ images before, 34, and 72 s after infusion of SPIO at a rate of 0.15 ml/min. **(b)** Dynamic signal changes of the two ROIs marked in **(a)**. **(c)** Contrast enhancement map at 34 s after SPIO infusion. **(d)** Histogram analysis of pixel intensities in **(c)**, showing two Gaussian distributions (red lines). Blue arrow points to where a cut-off of−53.9% was applied to separate the two distributions. **(e)** Segmented map shows the area where the relative signal change was smaller than −53.9%. **(f)** Contrast enhancement map, **(g)** histogram analysis, and **(h)** segmented map (ΔS% > 31.4) at 5 min after i.p. injection of Gd. **(i)** Bar graph and **(j)** correlation analysis of the BBBO territory predicted by SPIO and that assessed using Gd (*n* = 4, mean ± SD). The histological analyses show the region with extravasation of Evans blue **(k)** and rhodamine **(l, m); (m)** shows the zoomed-in area indicated by the white square in **(l)** following mannitol injection.

The infusion of SPIO results in a drop of T2^*^ hypointensity on MRI, which immediately clears once the infusion is stopped. This T2^*^ hypointensity in the brain can be sampled by GE-EPI at a temporal resolution of 2 images each second, which allows precise temporo-spatial visualization of the parenchymal perfusion territory. Initially, our transcatheter SPIO contrast delivery was set at 0.05 ml/min, and in those animals in which no drop of T2^*^ intensity was observed, we increased the speed at increments 0.05 ml/min, until the contrast agent appeared on real-time MRI. There was no need to increase the speed over 0.15 ml/min, which resulted in satisfactory and reproducible brain perfusion, as visualized by a characteristic reduction in signal intensity for the duration of the injection bolus (Figures 1a,b). Notably, increasing the infusion speed to 0.2 ml/min resulted in delayed brain injury, as shown by an abnormal signal on T2-weighted images and pathological changes on histology (Supplemental Figure 1). In addition, the injection rate for a satisfactory perfusion territory varied among mice, necessitating the titration of the injection speed for each animal.

### Real-time MRI for precise and local BBBO using mannitol

Immediately after the optimal infusion rate was determined for a particular mouse using SPIO, IA mannitol was infused at that rate for 1 min. To clearly present the MRI images, the signal change maps of SPIO-perfusion and Gd-contrast enhancement (Gd-CE) were calculated first (Figures 1c,f). As a consequence, such an approach resulted in an effective BBBO as reflected by gadolinium enhancement on the T1-weighted scan, which showed hyperintensity in the region previously highlighted by the contrast infusion (Figures 1c,f). The correlation between the SPIO-perfusion (Figure 1c) and Gd-CE (Figure 1f) MRI was determined. The histograms were drawn and fitted into two Gaussian distributions (Figures 1d,g). The values that corresponded to the minimal overlap between the two Gaussian functions were chosen to be the threshold that separated the pixels with a significant signal change. Using these determined thresholds, the areas with a significant signal change were determined (Figures 1e,h). For the four mice studied, the SPIO perfusion MRI showed an average signal change area of 26.00 ± 5.60%, while Gd-CE showed an average signal change area of 26.52 ± 5.33%, which was not significantly different (*P* = 0.829, Figure 1i). A good correlation was shown between these two methods (*r* = 0.937, *R*^2^ = 0.879, Figure 1j). This indicated a successful BBBO by IA mannitol, as predicted by the perfusion pre-scan. Furthermore, the histopathological validation using Evans Blue, which is a gold standard for BBB assessment and rhodamine, which was used as a surrogate marker of therapeutic agents, demonstrated a pattern of extravasation that was consistent with MRI (Figures 1k–m).

### Safety and long-term consequences of IA mannitol-induced BBBO

**Figure 2:**
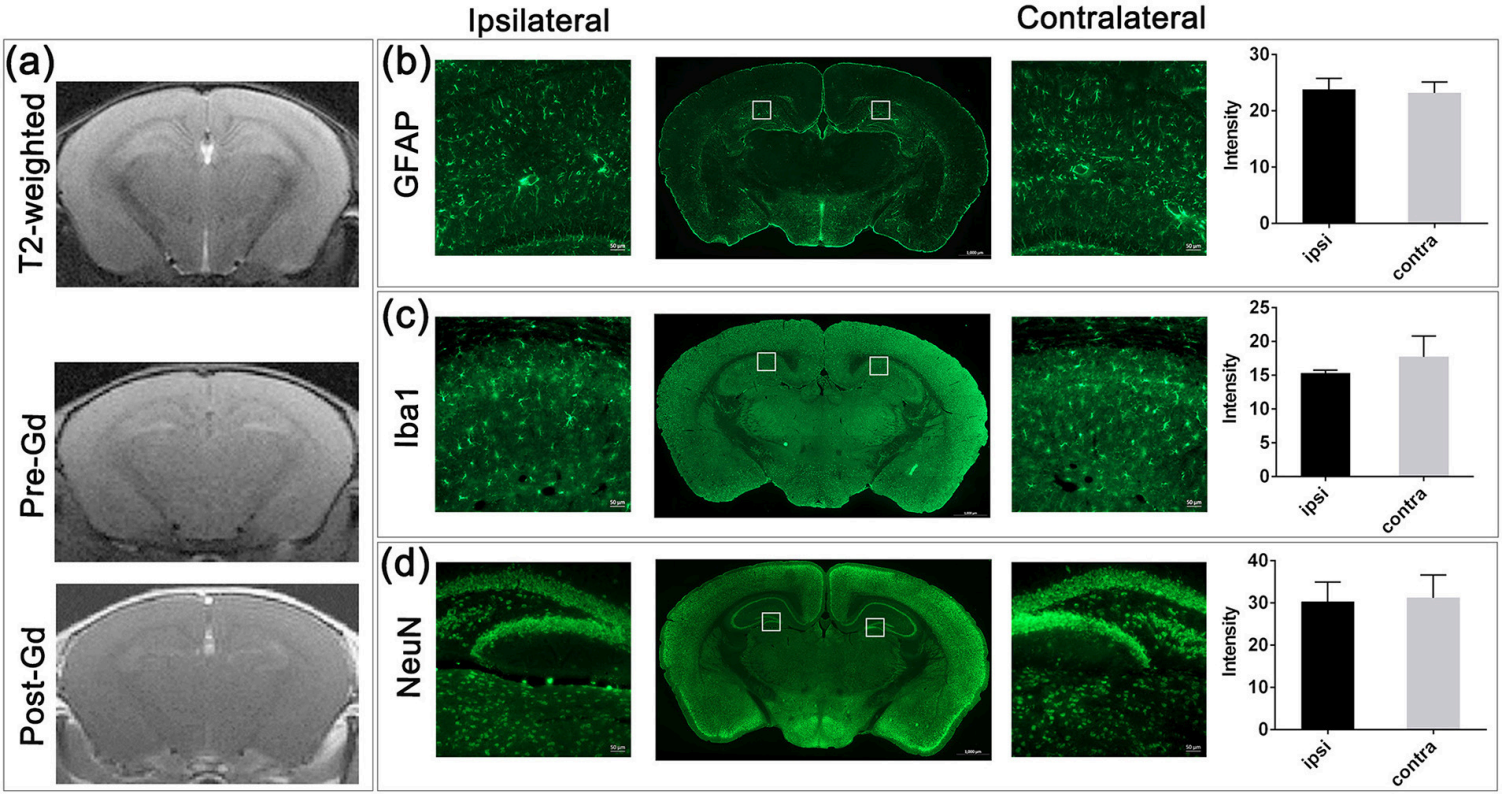
MRI and histological assessment post-BBBO. **(a)** T2-weighted, pre-Gd, and post-Gd images 3 days after BBBO showed no sign of brain damage. No Gd-CE could be observed in the brain, suggesting that the BBB was resealed. Fluorescent staining of the BBBO region with GFAP **(b)**, Iba1 **(c)**, and NeuN **(d)** revealed comparable intensity between the ipsilateral and the contralateral hemisphere (2 ROIs/hemisphere as represented in lower magnification, *n* = 4, mean ± SD) indicating no inflammation and no neuronal loss after BBBO.

To assess the safety of our BBBO protocol, mice were assessed for neurological and MRI sequelae. Three days after BBBO, MRI showed neither T2 abnormalities nor T1 Gd-enhancement (Figure 2a), indicating that the procedure was safe and that the BBB breach was transient, and did not cause permanent brain damage. Histology confirmed these observations with GFAP and IBA-1 staining 7 days post BBBO, in which there was no elevated astrocytic or microglia activation in the BBBO region, as measured by fluorescence intensity. There was no statistically significant difference in fluorescence intensity between the targeted region and the contralateral hemisphere (*P* = 0.571, *P* = 0.093; Figures 2b,c). Similarly, there was no evidence of neuronal damage based on NeuN staining (*P* = 0.331, Figure 2d).

## Discussion

The overall goal of BBBO is to maximize CNS targeting of the therapeutic agent while minimizing systemic toxicity. Various methods and drugs have been developed to induce transient permeabilization of the BBB, with IA mannitol-mediated osmotic disruption being the most frequently used procedure in both preclinical and clinical studies (7, 8, 21). Although osmotic BBBO has been an established method for decades, the parameters for inducing BBBO are highly variable and inconsistent. The infusion speed, in particular, is one of the most critical parameters in small animals and many published reports recommend an infusion that highly exceeds the physiological perfusion rate in the carotid artery, leading to brain damage (17, 22, 23). For example, in different preclinical studies, the infusion velocity of mannitol into the carotid artery for some rat studies was as low as 3.0 ml/min (14) or as high as 7.2 ml/min (13). Similarly, one mouse BBBO study reported that the procedure was performed with an IA infusion at a very high rate of 1.0 ml/min (6), which, in addition to the effect of mannitol, would likely have a direct damaging effect on the BBB. In that study, the PPA was not ligated, which might have added variability to the procedure, as the majority of the flow might be through the PPA and not the ICA (24). Indeed, we have shown that IA infusion into the rat internal carotid artery at rates exceeding 0.9 ml/min is damaging and results in scattered white matter hyperintensities (17). Here, we also found severe damage when the speed reached 0.2 ml/min indicating fine balance effective BBBO and damage. We also believe that PPA obstruction is necessary to route the entire contrast agent and mannitol volume to the cerebral arteries. Thus, we exploited dynamic susceptibility contrast MRI for perfusion prediction prior to BBBO. We escalated the rate from a low speed, increasing in 0.05 ml/min increments until the desired perfusion territory was reached. The extravasation of rhodamine and Evans blue corroborated the efficacy of BBBO, therefore suggesting that IA mannitol-mediated local BBBO is predictable and can be targeted to a specific region. The IA route for BBBO also allows immediate IA delivery of a specific drug during the same procedure, thus providing a “one-stop-shop” and improving the probability to achieve an adequate therapeutic concentration. Finally, SPIO in our study has been used at about 0.6 mg/kg which is similar to the dose previously used in patients (25). Hence, our procedure may have potential for future clinical translation.

## Author contributions

CC, GL, MJ, JB, SL, MP and PW conception and design. CC, GL and PW acquisition of data. CC, GL, MJ, JB, SL, MP and PW analysis and interpretation of data. CC draft of the manuscript. CC, GL, MJ, JB, SL, MP and PW revision, and final approval.

### Conflict of interest statement

The authors declare that the research was conducted in the absence of any commercial or financial relationships that could be construed as a potential conflict of interest.
