# Supplementary material for: Real-Time MRI Guidance for Reproducible Hyperosmolar Opening of the Blood-Brain Barrier in Mice
Source: Front Neurol. 2018 Oct 26;9:921. doi: 10.3389/fneur.2018.00921 (PMC6212512; doi:10.3389/fneur.2018.00921)
Supplement: Supplementary file 1 [file Data_Sheet_1.DOCX]

Supplementary Material

Real-time MRI guidance for precise hyperosmolar opening of the blood-brain barrier in mice

Chengyan Chu^1,2,3^, Guanshu Liu^1,6^, Miroslaw Janowski^1,2,4,5^, Jeff WM. Bulte^1,2^, Shen Li^1,2,3^, Monica Pearl^7^, Piotr Walczak^1,2,8*^

*** Correspondence:** Piotr Walczak, MD/PhD: pwalczak@mri.jhu.edu

# Supplementary Figure


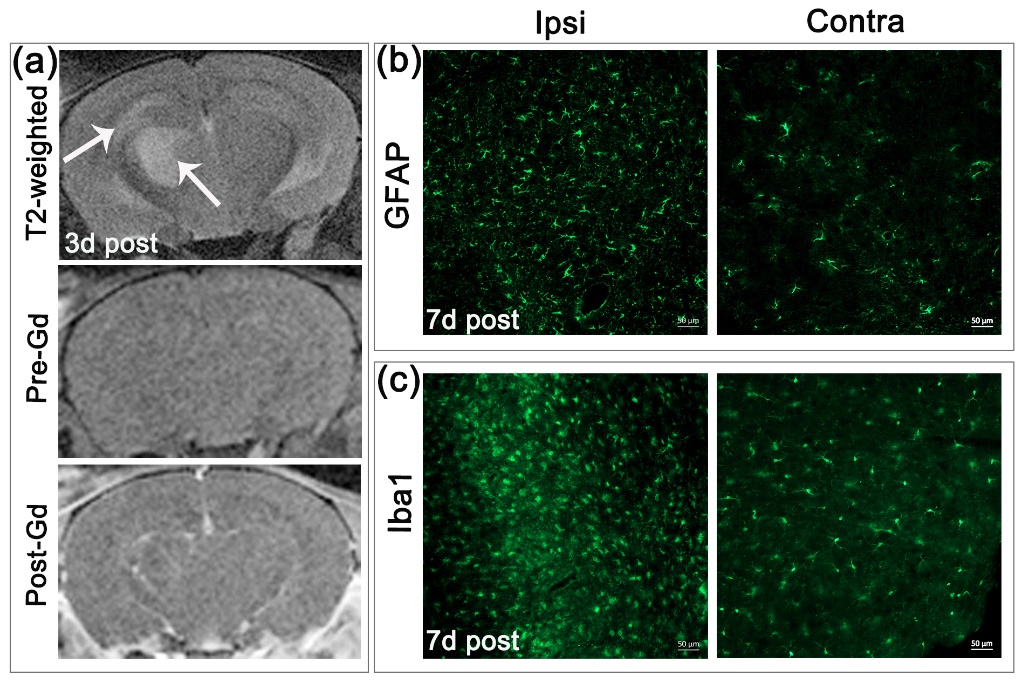


**Supplementary Figure 1. MRI and histological assessment post BBBO upon infusion of mannitol at high speed.** (**a**)**.** T2-weighted, pre-Gd, and post-Gd images three days post BBBO using a high-speed infusion rate (0.2 ml/min). The occurrence of brain damage was evident by the presence of a hyperintense region (arrow). Seven days later, astrocyte (GFAP) (**b**) and microglial activation (Iba1) (**c**) was evident in the area subjected to BBBO.
